# Efficacy and safety of metformin plus low-dose temozolomide in patients with recurrent or refractory glioblastoma: a randomized, prospective, multicenter, double-blind, controlled, phase 2 trial (KNOG-1501 study)

**DOI:** 10.1007/s12672-023-00678-3

**Published:** 2023-06-06

**Authors:** Wan-Soo Yoon, Jong Hee Chang, Jeong Hoon Kim, Yu Jung Kim, Tae-Young Jung, Heon Yoo, Se-Hyuk Kim, Young-Cho Ko, Do-Hyun Nam, Tae Min Kim, Se Hoon Kim, Sung-Hae Park, Youn Soo Lee, Hyeon Woo Yim, Yong-Kil Hong, Seung Ho Yang

**Affiliations:** 1 Department of Neurosurgery, Incheon St. Mary’s Hospital, College of Medicine, The Catholic University of Korea, Seoul, Korea; 2 Department of Neurosurgery, Severance Hospital, Yonsei University College of Medicine, Seoul, Korea; 3 Department of Neurological Surgery, Asan Medical Center, University of Ulsan College of Medicine, Seoul, Korea; 4 Department of Internal Medicine, Seoul National University Bundang Hospital, Seoul National University College of Medicine, Seongnam, Korea; 5 Department of Neurosurgery, Chonnam National University Hwasun Hospital, Hwasun, Korea; 6 Department of Neuro-Oncology Clinic, Center for Specific Organs Cancer, National Cancer Center Hospital, National Cancer Center, Goyang, Korea; 7 Department of Neurosurgery, Ajou University Hospital, Ajou University School of Medicine, Suwon, Korea; 8 Department of Neurosurgery, Konkuk University Medical Center, Seoul, Korea; 9 Department of Neurosurgery, Samsung Medical Center, Sungkyunkwan University School of Medicine, Seoul, Korea; 10 Department of Internal Medicine, Seoul National University Hospital, Seoul National University College of Medicine, Seoul, Korea; 11 Department of Pathology, Severance Hospital, Yonsei University College of Medicine, Seoul, Korea; 12 Department of Pathology, Seoul National University Hospital, Seoul, Korea; 13 Department of Hospital Pathology, Seoul St. Mary’s Hospital, College of Medicine, The Catholic University of Korea, Seoul, Korea; 14 Department of Preventive Medicine, College of Medicine, The Catholic University of Korea, Seoul, Korea; 15 Department of Neurosurgery, Hallym University Sacred Heart Hospital, The Hallym University Medical Center, 22, Gwanpyeong-ro 170 beon-gil, Dong-gu, Anyang-si, Gyeongggi-do, 14068 Korea; 16 Department of Neurosurgery, St. Vincent’s Hospital, College of Medicine, The Catholic University of Korea, 93 Jungbudaero, Paldal-gu, Suwon, Seoul, 16247 Korea

**Keywords:** Glioblastoma, Metformin, Temozolomide, Clinical study, Therapeutics, Survival

## Abstract

**Purpose:**

Glioblastoma (GBM) has a poor prognosis after standard treatment. Recently, metformin has been shown to have an antitumor effect on glioma cells. We performed the first randomized prospective phase II clinical trial to investigate the clinical efficacy and safety of metformin in patients with recurrent or refractory GBM treated with low-dose temozolomide.

**Methods:**

Included patients were randomly assigned to a control group [placebo plus low-dose temozolomide (50 mg/m^2^, daily)] or an experimental group [metformin (1000 mg, 1500 mg, and 2000 mg per day during the 1st, 2nd, and 3rd week until disease progression, respectively) plus low-dose temozolomide]. The primary endpoint was progression-free survival (PFS). Secondary endpoints were overall survival (OS), disease control rate, overall response rate, health-related quality of life, and safety.

**Results:**

Among the 92 patients screened, 81 were randomly assigned to the control group (43 patients) or the experimental group (38 patients). Although the control group showed a longer median PFS, the difference between the two groups was not statistically significant (2.66 versus 2.3 months, *p* = 0.679). The median OS was 17.22 months (95% CI 12.19–21.68 months) in the experimental group and 7.69 months (95% CI 5.16–22.67 months) in the control group, showing no significant difference by the log-rank test (HR: 0.78; 95% CI 0.39–1.58; *p* = 0.473). The overall response rate and disease control rate were 9.3% and 46.5% in the control group and 5.3% and 47.4% in the experimental group, respectively.

**Conclusions:**

Although the metformin plus temozolomide regimen was well tolerated, it did not confer a clinical benefit in patients with recurrent or refractory GBM.

*Trial registration* NCT03243851, registered August 4, 2017.

**Supplementary Information:**

The online version contains supplementary material available at 10.1007/s12672-023-00678-3.

## Introduction

Glioblastoma (GBM) is the most common tumor of primary malignancy in the brain and spine. Current treatment options for GBM include surgical resection, radiotherapy, and concomitant and/or adjuvant chemotherapy. However, GBM is a fatal disease with a poor prognosis [1]. Although many clinical studies have been conducted in recent decades, Stupp’s protocol with concurrent chemoradiotherapy (CCRT) using temozolomide (TMZ) is considered the standard treatment for GBM [2–4]. The best treatment for recurrent or refractory GBM has not yet been established. Many clinical studies of patients with recurrent or refractory GBM are currently being conducted [5–7]. Among them, low-dose daily temozolomide is one of the suggested treatment options for patients with recurrent GBM, as it is well tolerated and has acceptable toxicity [8, 9].

Metformin (N,N-dimethylbiguanide) is a biguanide antidiabetic medication. It has been suggested as the first-line medication for patients with type 2 diabetes mellitus (DM). It can inhibit mitochondrial complex I, lower cellular adenosine triphosphate (ATP) levels, cause adenosine monophosphate (AMP)-activated protein accumulation, and disrupt downstream cAMP-protein kinase A (PKA) signaling, resulting in inhibition of glycolysis in the liver [10]. Lower systemic glucose and insulin levels by metformin use might decrease insulin-mediated tumor growth and progression in cancer. In addition, metformin can activate 5’-AMP-activated protein kinase (AMPK) to restrain cell growth and proliferation. AMPK can inhibit AKT and mTORC1 signaling, activate HIF-1α, p53, cMYC, and DICER1, and suppress fatty acid synthesis [11]. The inhibitory effect of metformin via activated AMPK suggests that metformin is a potential therapeutic agent in cancer. Randomized controlled trials have reported the clinical effects of metformin on the survival prognosis of patients with lung and pancreatic cancer [12, 13]. However, only a few trials have reported a benefit of add-on metformin to standard therapy due to inadequate dosages, limitations associated with observational studies, and assessment of patients with advanced disease.

The antitumor effect of metformin on glioma cells has been suggested by in vitro and in vivo murine models [14, 15]. However, the therapeutic implication of metformin use in patients with GBM is controversial due to a lack of well-designed clinical trials [16]. In this study, we performed the first randomized, prospective, phase 2 trial to investigate the clinical efficacy and safety of metformin in patients with recurrent or refractory GBM treated with low-dose temozolomide.

## Materials and methods

### Patient selection

This study was planned as a prospective, multicenter, phase 2 trial by the Korean Neuro-Oncology Group of the Korean Society for Neuro-Oncology (KSNO). Patients were randomized into two groups, a control group and an experimental group, with a double-blind process by a central agency. The trial was registered with ClinicalTrials.gov (NCT03243851). It was conducted at twelve tertiary hospitals in the Republic of Korea.

GBM patients with recurrence or progression after surgery, radiotherapy, chemotherapy, and/or targeted therapy were eligible for inclusion. Additional inclusion criteria were as follows: age of at least 19 years; Karnofsky performance score (KPS) ≥ 60; disease progression on MRI as defined by the Response Assessment in Neuro-Oncology (RANO) criteria [16] at least 4 weeks after completion of operation or chemotherapy; bone marrow function test results (hemoglobin > 9.0 g/dL, white blood cells > 3.0 × 10^9^/L, absolute neutrophil count > 1500 per mm^3^ without transfusion or granulocyte colony-stimulating factor, platelet count ≥ 100,000 per µL), liver function (total bilirubin < 1.5 × upper limit of normal [ULN], alanine aminotransferase and aspartate aminotransferase < 2.5 × ULN); and renal function test results (serum creatinine ≤ 1.5 mg/dL). Assessment of O6-methylguanine-DNA methyltransferase (MGMT) methylation status at first surgery was also needed. The exclusion criteria were pregnancy or breastfeeding; leptomeningeal metastasis; diabetes; hypersensitivity or intolerance to metformin; patients who were currently taking thiazolidinediones, sulfonylureas, metformin, and insulin regardless of the reason; congestive heart failure, unstable angina or incomplete arrhythmia, uncontrolled hypertension despite optimal medical management; myocardial infarction within 6 months before the start of drug administration; cerebrovascular accident; or uncontrolled infection.

### Study design and treatment scheme

Screened patients were enrolled according to a randomization sequence created by an independent statistician using the PROC PLAN procedure of SAS 9.4 with a 1:1 allocation using block randomization. This allocation was implemented by the central agency. Each enrolled patient was assigned an encrypted trial number. Data analysts and outcome assessors were blinded. Statisticians remained blinded to treatment allocation until all data had been analyzed to minimize bias.

Patients randomly assigned to the control (placebo plus low-dose temozolomide) or experimental (metformin plus low-dose temozolomide) group were administered medication daily. Placebo or metformin was given at 1000 mg/day for the first week, at 1500 mg/day for the second week, and at 2000 mg/day from the third week until death, disease progression, unacceptable toxicity, or withdrawal of consent. Simultaneously, patients were administered low-dose temozolomide (50 mg/m^2^) daily. To manage toxicity, prespecified dose modifications were planned in the protocol. Patients requiring discontinuation and dose reduction of metformin could have their dose raised again at the discretion of the investigator once toxicity had been resolved to baseline levels. Treatment was discontinued if toxicity did not reverse after 4 weeks of discontinuation.

Serum chemistry and hematological parameters were evaluated every 4 weeks. Radiological evaluations were performed with gadolinium-enhanced brain MRI every 8 weeks from the first drug administration until disease progression (or within one week on either side of this time point). Responses were assessed by each institutional investigator according to the RANO criteria [17]. The MGMT methylation status of tumor tissues was assessed in the laboratory of each participating center using methylation-specific polymerase chain reaction. Safety evaluations were performed from the start of treatment until 30 days after the last dose of study drug and included adverse events, serum laboratory findings, vital signs, and electrocardiography. Adverse events were graded using the National Cancer Institute Common Terminology Criteria for Adverse Events, version 4.0. Health-related quality of life (QoL) was measured using the European Organization for Research and Treatment of Cancer quality-of-life questionnaire (QLQ-C30) and brain module (QLQ-BN20) questionnaires administered concurrently with MRI evaluation.

### Endpoints of study

The primary endpoint of the study was progression-free survival (PFS) time, which was defined as the time from randomization to tumor progression based on the RANO criteria or to death. The secondary endpoint was overall survival (OS), which was the time from randomization to death due to any cause. The proportion of patients in whom disease control was achieved, including stable disease, partial response, and complete response, and the proportion with a good response, including partial or complete response, were also evaluated as secondary endpoints. Additionally, the health-related QoL and safety of patients were assessed as secondary endpoints. Radiological and clinical findings were evaluated by each institutional investigator and supervised by the central agency.

### Statistical analysis

The primary endpoint of PFS was used to determine the sample size for the study based on the following assumptions: a two-sided log-rank test at the 0.1 level of significance, 80% power to detect a hazard ratio (HR) for the experimental group versus the control group of 0.58 corresponding to 25% control group and 45% experimental group at 6 months for PFS. With an anticipated accrual period of 18 months, a follow-up of 6 months, and a 10% dropout, we calculated that at least 54 patients per group (a total of 108 patients) were needed. Sample size calculations were performed using PASS 13 software (PASS Institute Inc. Kaysville, Utah). The intention-to-treat (ITT) population comprised all randomized patients by their assigned treatment arms, whereas the per-protocol (PP) population was composed of ITT patients who had received at least one dose of study therapy without any major protocol deviations. We first performed primary efficacy analysis on the ITT population for primary and secondary endpoints. Sensitivity analyses were performed for the PP population. The PP population was also used for safety analyses.

The primary end point for PFS was defined as the time from randomization to progression or death, whichever occurred first. Censoring rules for PFS followed those described in the 2007 FDA guidance on clinical trial endpoints. OS was defined as the duration between the date of randomization and death or censoring at the date of the last follow-up. We estimated PFS and OS using the Kaplan–Meier method with the median survival duration summarized. The log-rank test was used to compare the control and experimental groups. The pointwise 95% confidence interval (CI) of the overall survival rate or progression-free survival rate for 6, 12, and 24 months was calculated with Greenwood’s formula using complementary log-log transformation. Hazard ratios (HRs) and 95% confidence intervals (CIs) for PFS and OS were estimated using the Cox proportional hazard model. Proportional hazards assumptions were confirmed by Schoenfeld residuals and Supremum tests.

Response rates were compared between groups using the chi-square test or Fisher’s exact test when appropriate. We evaluated and tabulated the number of patients with at least one reported adverse event by the group. The effects of treatments on QoL were analyzed by generalized estimating equation (GEE) models with an unstructured correlation structure, normal distribution, and identity link function. A GEE model included treatment, time, and the interaction term between treatment and time. The results were regarded as statistically significant when the p value was less than 0.05. All statistical analyses were performed using SAS ver. 9.4 (SAS Institute Inc., Cary, NC, USA).

### Ethical statement

A total of 12 tertiary hospitals in the Republic of Korea participated in this study, and ethical approval was received from each institutional review board before enrollment began. All patients provided written informed consent in accordance with national guidelines. Patients were not compensated for their participation.

## Results

### Patient characteristics

**Fig. 1:**
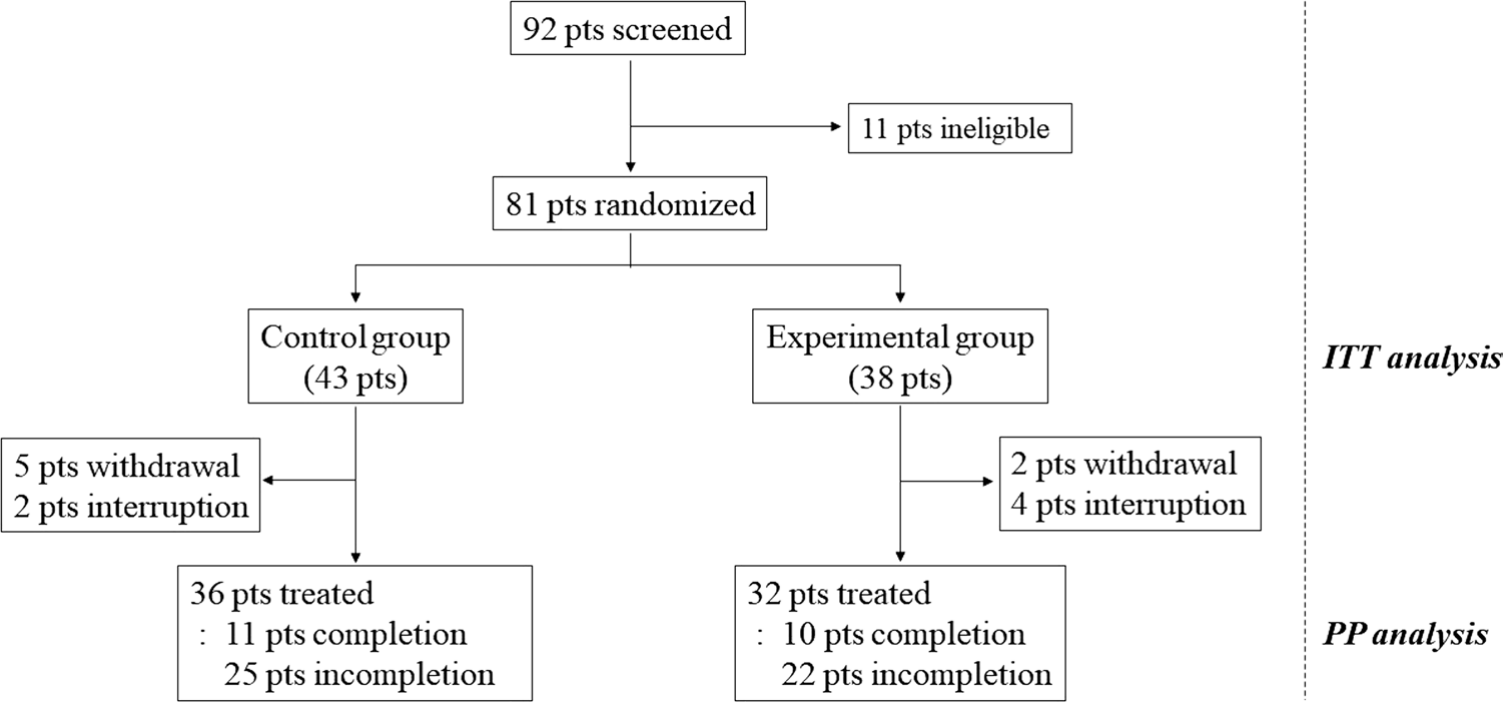
Study flow diagram representing patient selection and randomization. The intention-to-treat (ITT) analysis included all randomized patients, and the per-protocol (PP) analysis was performed for the treated patients

Between Dec. 14, 2016, and Aug. 20, 2020, 92 patients were screened, and 81 eligible patients were randomly assigned to a control group (n = 43) or an experimental group (n = 38). Among the 43 patients assigned to the control group, the study was interrupted for 7 patients because of withdrawal of consent (n = 5) or medical problems (n = 2). The remaining 36 patients in the control group were treated with the scheduled protocol. Eleven patients completed six cycles of intervention, whereas 25 patients did not complete treatment due to disease progression (n = 20), side effects (n = 3), cerebral infarction (n = 1), or denial of treatment (n = 1). Among the 38 patients assigned to the experimental group, the study was stopped for 6 patients because of withdrawal of consent (n = 2) or deterioration of the patient’s condition (n = 4) before the clinical trial. The remaining 32 patients in the experimental group followed the treatment schedule. Twenty-eight patients received up to 2000 mg/day of metformin. Ten patients completed the intervention, whereas 22 patients did not complete treatment because of disease progression (n = 21) or side effects (n = 1) (Fig. 1). For baseline characteristics, we performed descriptive statistics for both the ITT and PP populations. Because there was no characteristic difference between the ITT and PP populations, the ITT population was analyzed for primary and secondary endpoints. Clinical characteristics such as median age, sex ratio, height, body weight, and KPS were not significantly different between the two treatment groups. However, patients in the control group had a higher frequency of MGMT methylation status than those in the experimental group (42.9% versus 21.6%, *p* = 0.045) (Table 1).

**Table 1:** Patient characteristics of ITT and PP population

ITT population				PP population			
	Control group (n = 43)	Experimental group (n = 38)	*p-value*		Control group (n = 36)	Experimental group (n = 32)	*p-value*
Patient demographic				Patient demographic			
Age (years)				Age (years)			
Mean ± sd	55.5 ± 11.8	55.9 ± 10.6	0.992	Mean ± sd	55.3 ± 12.6	55.1 ± 10.9	0.64
Median (IQR)	57.0 (48.0–64.0)	56.5 (50.0–63.0)		Median (IQR)	57.5 (46.0-64.5)	55.5 (49.5–61.0)	
<65	33 (76.7)	32 (84.2)	0.4	<65	27 (75.0)	28 (87.5)	0.191
≥65	10 (23.3)	6 (15.8)		≥65	9 (25.0)	4 (12.5)	
sex				sex			
Male	21 (48.8)	22 (57.9)	0.415	Male	17 (47.2)	20 (62.5)	0.207
Female	22 (51.2)	16 (42.1)		Female	19 (52.8)	12 (37.5)	
Physical examination				Physical mxamination			
Height (cm)				Height (cm)			
Mean ± sd	164.7 ± 9.6	164.6 ± 11.2	0.773	mean ± sd	164.5 ± 9.2	165.0 ± 11.8	0.522
Median (IQR)	163.0 (157.0-173.0)	166.5 (156.0-173.0)		median (IQR)	163.0 (157.0-172.5)	170.0 (157.5–174.0)	
Weight (kg)				Weight (kg)			
Mean ± sd	62.6 ± 12.4	68.0 ± 12.9	0.052	mean ± sd	62.4 ± 11.7	69.2 ± 13.4	0.038
Median (IQR)	62.0(52.0–73.0)	66.0(59.0–81.0)		median (IQR)	63.0(52.0–73.0)	69.5(59.5–81.0)	
Karnofsky Performance Status (KPS)				Karnofsky performance status (KPS)			
60	9 (20.9)	4 (10.5)	0.103	60	8 (22.2)	2 (6.3)	0.18
70	4 (9.3)	11 (28.9)		70	4 (11.1)	9 (28.1)	
80	14 (32.6)	9 (23.7)		80	12 (33.3)	9 (28.1)	
90	12 (27.9)	13 (34.2)		90	10 (27.8)	11 (34.4)	
100	4 (9.3)	1 (2.6)		100	2 (5.6)	1 (3.1)	
MGMT methylation				MGMT methylation			
No	24 (57.1)	29 (78.4)	0.045	No	19 (54.3)	24 (77.4)	0.049
Yes	18 (42.9)	8 (21.6)		Yes	16 (45.7)	7 (22.6)	

* ITT* intention-to-treat; *PP* per-protocol population; *sd* standard deviation; *IQR* interquartile range; *cm* centimeters; *kg* kilograms; *MGMT* O6-methylguanine-DNA methyltransferase

### Progression-free survival, overall survival, and overall radiological response

**Fig. 2:**
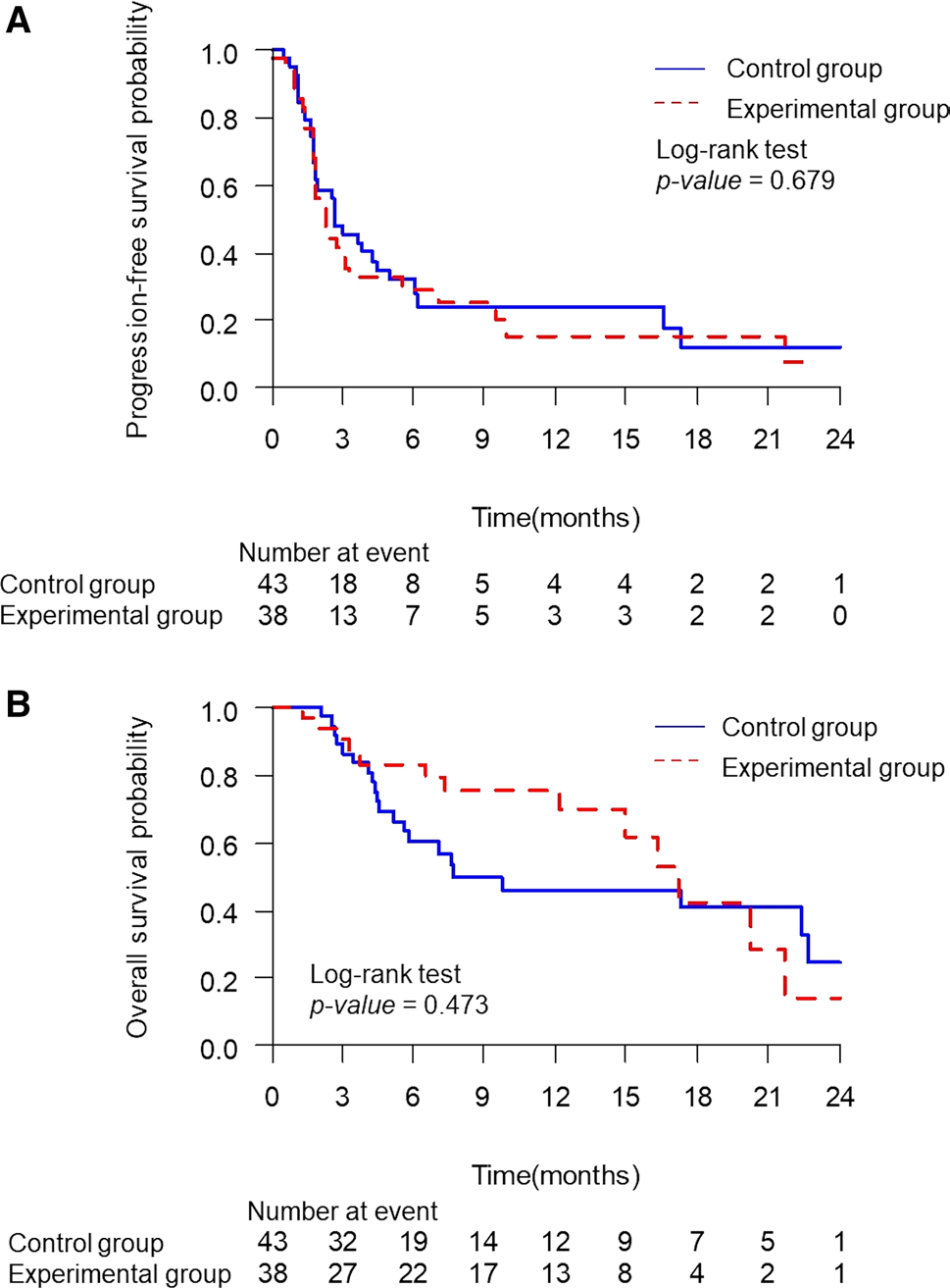
Progression-free survival (**A**) and overall survival (**B**) in all patients. There were no significant differences between the control and experimental groups in survival outcomes (p = 0.679 and 0.473 in PFS and OS, respectively). *PFS* progression-free survival; *OS* overall survival

At the analysis cutoff date, 59 patients showed disease progression or death: 31 (72.1%) of 43 in the control group and 28 (73.7%) of 38 in the experimental group. The median PFS time was 2.66 months (95% CI 1.74–4.40 months) in the control group and 2.3 months (95% CI 0.67–1.87 months) in the experimental group. The PFS time between the two groups was not significantly different by the log-rank test (HR: 1.12; 95% CI 0.67–1.87; *p* = 0.679). PFS at 6 and 12 months was 32% (95% CI 0.18–0.47) and 24% (95% CI 0.11–0.40) in the control group and 29% (95% CI 0.15–0.45) and 15% (95% CI 0.04–0.32) in the experimental group, respectively. OS tended to be improved in the experimental group compared with the control group. The median OS was 17.22 months (95% CI 12.19–21.68 months) in the experimental group and 7.69 months (95% CI 5.16–22.67 months) in the control group, showing no significant difference by the log-rank test (HR: 0.78; 95% CI 0.39–1.58; *p* = 0.473). OS at 6 and 12 months was 83% (95% CI 0.64–0.93) and 75% (95% CI 0.55–0.88) in the experimental group and 60% (95% CI 0.42–0.74) and 46% (95% CI 0.28–0.62) in the control group, respectively (Table 2; Fig. 2).

**Table 2:** Progression-free survival and overall survival of patient subgroup

	Control group (n = 43)	Experimental group (n = 38)	*p-value*	HR (95% CI)
Progression free survival				
Progression or death (%)	31 (72.1)	28 (73.7)		
At 6 month PFS rate (95% CI)	0.32 (0.18–0.47)	0.29 (0.15–0.45)		
At 12 month PFS rate (95% CI)	0.24 (0.11–0.40)	0.15 (0.04–0.32)		
At 24 month PFS rate (95% CI)	0.12 (0.03–0.29)	N/A		
Median PFS time in months (95% CI)	2.66 (1.74–4.40)	2.30 (1.74–3.29)	0.679	1.12 (0.67–1.87)
Overall survival				
Death (%)	21 (48.8)	13 (34.2)		
At 6 month OS rate (95% CI)	0.60 (0.42–0.74)	0.83 (0.64–0.93)		
At 12 month OS rate (95% CI)	0.46 (0.28–0.62)	0.75 (0.55–0.88)		
At 24 month OS rate (95% CI)	0.25 (0.08–0.46)	0.14 (0.01–0.44)		
Median OS time in months (95% CI)	7.69 (5.16–22.67)	17.22 (12.19–21.68)	0.473	0.78 (0.39–1.58)

HR (95% CI) were obtained from the Cox proportional hazards regression models. Survival rates and median time were estimated from Kaplan-Meier analyses.

*HR* hazard ratio; *CI* confidence interval; *PFS* progression-free survival; *OS* overall survival

After study completion, the overall radiological response of the control group was CR in 2 patients, PR in 2, SD in 16, and PD in 16. On the other hand, there were 2 patients with PR, 16 with SD, and 15 with PD in the experimental group. The disease control rate and the good response rate in the control versus experimental groups were 46.5% and 9.3% versus 47.4% and 5.3%, respectively. They were not significantly different between the two groups (*p* = 0.939 for disease control rate and *p* = 0.679 for good response rate, Table 3).

**Table 3:** The response rate of treatment in the patient subgroup

	Control group (n = 43)	Experimental group (n = 38)	*p-value*
Overall radiologic response (%)			0.808
CR	2 (4.7)	0 (0.0)	
PR	2 (4.7)	2 (5.3)	
SD	16 (37.2)	16 (42.1)	
PD	16 (37.2)	15 (39.5)	
Not Assessed	7 (16.3)	5 (13.2)	
Disease control (%)	20 (46.5)	18 (47.4)	0.939
(CR, PR, or SD)			
Good response (%)	4 (9.3)	2 (5.3)	0.679
(CR or PR)			

P-values are calculated by Chi-square, Fisher’s exact test

*CR* complete response; *PR* partial response; *SD* stable disease; *PD* progression of disease.

### Adverse events

Treatment-related side effects were evaluated for patients who had received at least one dose of study therapy in the PP population. One patient in the control group showed sudden cardiac arrest to death, and one patient had acute cerebral infarction during treatment. Two patients showed treatment-related grade 3 adverse effects, including anemia and hypertension. However, there was no grade 3 toxicity event in the experimental group. Grade 2 adverse effects were observed in 15 and 6 patients in the control group and experimental group, respectively. Common symptoms were fatigue and diarrhea (Table 4).

**Table 4:** Adverse events of the patient subgroup

	Control group					Experimental group	
	Grade 2	Grade 3	Grade 4	Grade 5	Grade 2		Grade 3
Laboratory abnormalities							
Anemia	1	1	None	none	None		None
Increased ALT/AST	2	None	None	none	None		None
Clinical adverse events							
Abdominal pain	1	None	None	None	None		None
Cardiac arrest	None	None	None	1	None		None
Cerebral infarction	None	None	1	None	None		None
Constipation	2	None	None	None	None		None
Diarrhea	1	None	None	None	3		None
Dyspepsia	1	None	None	None	None		None
Fatigue	5	None	None	None	None		None
Headache	None	None	None	None	1		None
Hypertension	1	1	None	None	2		None
Lethargy	1	None	None	None	None		None

*ALT* Alanine aminotransferase; *AST* Aspartate aminotransferase

### Quality of Life

To evaluate QoL questionnaires, the longitudinal development of each dimension compared with baseline and each visit time was analyzed using a GEE model. The global health score was higher (between 8 and 12 weeks) in the control group. Physical function, role function, and emotional function were similar between the two groups except that the control group showed better results at a visit time of 12 weeks. Cognitive function and social function were also similar between the two groups. In symptom scales, fatigue, nausea/vomiting, pain, insomnia, appetite loss, constipation, diarrhea, and financial impact were similar. Dyspnea was higher in the experimental group at 12 weeks. For the BN20 symptom scales, future uncertainty, communication deficit, seizure, drowsiness, hair loss, itchy skin, and weakness of legs were similar. Visual disorder and motor dysfunction were higher in the experimental group at 8 and 12 weeks, respectively. Headache was higher in the control group at 8 weeks. Bladder control was higher in the control group at 16 weeks. The results of the QoL questionnaires according to the time flow are summarized in Supplementary Table 1.

## Discussion

In vitro and experimental in vivo studies have demonstrated the antitumor effect of metformin on glioma cells, including human and rat glioma cells. The most proposed mechanism involved in this effect of metformin is the activation of AMPK. AMPK is a metabolic tumor suppressor that maintains cell metabolism and growth at appropriate levels. It can also activate the response to energy stress in the microenvironment of tumors. AMPK can act on mTOR, p53, and fatty acid synthase, resulting in suppression of cell proliferation and growth [18]. Two antidiabetic drugs, metformin and thiazolidinediones, are known to activate AMPK [19]. In a glioma cell line, metformin can decrease mitochondrial-dependent ATP production and oxygen consumption and increase lactate and glycolytic ATP production. It can also induce anti-proliferation, autophagy, apoptosis, and cell death with AMPK and Redd1 activation in addition to mTOR pathway inhibition [20–23]. Metformin is also associated with the inhibition of AKT activation by downregulating phosphorylation [24, 25]. In another study, metformin repressed glioma proliferation through mTOR inhibition by increasing PRAS40, which is an AKT substrate that can bind to RAPTOR to negatively regulate mTOR. This was observed to be independent of AMPK [26].

Metformin also exhibits a cytotoxic effect on glioma stem cells mediated by inhibition of the AKT pathway [25, 27]. In another study, metformin inhibited glioma cell stemness and epithelial-mesenchymal transition via inhibition of YAP activity, a critical executor of the Hippo pathway [28]. Metformin can also inhibit glioma stem cell proliferation by G1 arrest. It is mediated by inhibition of chloride intracellular channel 1, which is progressively oxidized during cell cycle progression as a function of chloride selective ion channels. It is required for glioma stem cell proliferation [29, 30]. Inhibition of glioma cell invasion by metformin is another finding of an in vitro study [23]. Metformin can suppress the expression of matrix metalloproteinase-2, a key effector of glioma cell invasion, by downregulating fibulin-3 at the transcriptional level [31].

TMZ plus metformin shows a synergistic effect on glioma cells in vitro. Compared with TMZ or metformin monotherapy, this combination enhances AMPK phosphorylation and inhibits mTOR phosphorylation, AKT phosphorylation, and p53 expression [32, 33]. Synergistic reduction of gliosphere formation and expansion of glioma stem cells has also been investigated by this combination treatment, which was accompanied by AMPK activation [34]. Moreover, this TMZ and metformin combination has synergistic antitumor effects even in TMZ-resistant glioma cells [35–37].

The therapeutic effect of metformin in patients with malignant gliomas has been reported but remains controversial. A retrospective investigation was performed to determine the effects of diabetes mellitus (DM), corticosteroid therapy, and metformin therapy on progression and survival in 276 primary glioblastoma patients [38]. Despite the limitation that only 44 patients with DM and 20 patients treated with metformin were included, corticosteroid therapy and hyperglycemia were strongly associated with impaired survival rates. DM did not affect survival outcome. However, metformin therapy prolonged progression-free survival in GBM patients with DM [38]. In a systematic review of the relationship between type 2 DM and hyperglycemia in GBM patients, elevated BMI was found to be an independent risk factor for poor outcome and shorter OS. Among the 20 included papers, 4 studies with a total of 3003 patients evaluated the effect of metformin on survival in DM patients with GBM. Three studies including a total of 1272 patients showed better overall survival in the patients treated with metformin. However, another paper involving 1731 patients showed a null relationship between metformin and OS in GBM patients [16]. A retrospective cohort study of 231 patients with WHO grade III gliomas and 862 patients with WHO grade IV gliomas was performed to determine the survival of patients depending on metformin therapy. This study found that metformin was associated with significantly better overall and progression-free survival in patients with WHO grade III gliomas but not in those with WHO grade IV gliomas. Additionally, a relationship between IDH mutation and metformin sensitivity has been proposed [39].

A phase Ib clinical trial, the only clinical prospective study to the best of our knowledge, was conducted with metformin and chloroquine, an antimalarial drug with a putative anticancer function, for IDH-1-mutated solid cancers. This study included 12 cholangiocarcinoma, 2 glioma, and 3 chondrosarcoma patients in total. The combinational treatment was performed with a median duration of 43 days under a well-tolerated state, but it did not show a clinical benefit in this trial [40]. Similarly, adding metformin to standard chemotherapy failed to improve the clinical outcomes in patients with breast cancer, prostate cancer, and lung cancer [12, 41–44].

Our study is the first clinical study designed as a prospective, double-blind, randomized trial to investigate the clinical significance of metformin in patients with recurrent or progressive GBM. This study could not identify any survival benefit of metformin on PFS or OS in the included patients. Metformin had no survival advantage at the 3 time points (≤ 6 months, > 6-≤15 months and ≥ 15 months). The tendency of OS prolongation might be attributable to postprogression therapy at each institution. However, treatment with metformin at a maximal dosage of 2000 mg/day was safe and tolerated with minimal toxicity and well-preserved QoL in patients with GBM.

This study included a relatively limited number of patients with recurrent GBM. To minimize bias of patients by major protocol deviations, such as withdrawal of consent, this study was analyzed with an ITT method, including all randomized patients. However, 7 (16.3%) and 6 (15.8%) patients in the control and experimental groups, respectively, were interrupted before the treatment. It is possible that the difference in survival outcomes between the two groups has not been confirmed due to a decrease in the number of patients participating in clinical studies. Because the clinically recommended maximum dosage of metformin is 2000 mg/day, this study was designed with 1000 mg/day as the starting dose and 2000 mg/day as the maximum dose. However, it is also possible that this 2000 mg/day dosage of metformin is still insufficient to show a positive survival outcome. In future studies, it will be necessary to confirm the difference in survival outcomes based on metformin dosages including a dosage of over 2000 mg/day or based on a novel delivery system. Additionally, metformin can inhibit insulin-mediated tumor growth by lowering systemic insulin levels. However, systemic insulin levels were not measured in this study, so the survival outcome depending on insulin level by metformin was not assessed.

## Conclusion

Although metformin is well tolerated, our study did not show that metformin confers a benefit in survival outcomes or radiological response in patients with recurrent or refractory GBM.

## Electronic supplementary material

Supplementary Material 1

## Data Availability

The datasets generated and/or analyzed during the current study are not publicly available but are available from the corresponding author on reasonable request.
