# Supplementary material for: Efficacy and safety of metformin plus low-dose temozolomide in patients with recurrent or refractory glioblastoma: a randomized, prospective, multicenter, double-blind, controlled, phase 2 trial (KNOG-1501 study)
Source: Discov Oncol. 2023 Jun 6;14:90. doi: 10.1007/s12672-023-00678-3 (PMC10244311; doi:10.1007/s12672-023-00678-3)
Supplement: Supplementary file 1 — Supplementary Material 1 [file 12672_2023_678_MOESM1_ESM.docx]

Supplementary table 1. Summary descriptive statistics (mean, standard deviation) of quality of life

|  | baseline | | 4 week | | 8 week | | 12 week | | 16 week | | 20 week | | 24 week | |
| --- | --- | --- | --- | --- | --- | --- | --- | --- | --- | --- | --- | --- | --- | --- |
|  | n | mean ± sd | n | mean ± sd | n | mean ± sd | n | mean ± sd | n | mean ± sd | n | mean ± sd | n | mean ± sd |
| *C30 Global health status* |  |  |  |  |  |  |  |  |  |  |  |  |  |  |
| Control group | 39 | 59.2 ± 27.3 | 28 | 59.2 ± 23.1 | 21 | 67.1 ± 23.2 | 15 | 67.8 ± 23.5 | 14 | 54.2 ± 29.6 | 11 | 57.6 ± 37.5 | 21 | 58.7 ± 34.2 |
| Experimental group | 37 | 55.6 ± 22.4 | 26 | 47.8 ± 22.1 | 18 | 55.6 ± 19.0 | 12 | 58.3 ± 18.5 | 9 | 52.8 ± 26.4 | 9 | 61.1 ± 21.2 | 16 | 43.8 ± 18.1 |
| *C30 functional scales* |  |  |  |  |  |  |  |  |  |  |  |  |  |  |
| Physical function |  |  |  |  |  |  |  |  |  |  |  |  |  |  |
| Control group | 39 | 65.3 ± 30.7 | 28 | 62.4 ± 33.9 | 21 | 72.1 ± 31.5 | 15 | 83.6 ± 17.1 | 14 | 75.2 ± 27.7 | 11 | 81.8 ± 19.1 | 21 | 65.1 ± 35.2 |
| Experimental group | 37 | 57.3 ± 29.9 | 26 | 54.6 ± 33.6 | 18 | 64.4 ± 28.5 | 12 | 51.1 ± 31.1 | 9 | 62.2 ± 37.9 | 9 | 60.0 ± 37.0 | 16 | 49.6 ± 35.9 |
| Role function |  |  |  |  |  |  |  |  |  |  |  |  |  |  |
| Control group | 39 | 59.0 ± 38.8 | 28 | 62.5 ± 39.2 | 21 | 73.8 ± 34.0 | 15 | 84.4 ± 24.0 | 14 | 72.6 ± 34.3 | 11 | 80.3 ± 34.0 | 21 | 65.1 ± 38.3 |
| Experimental group | 37 | 51.8 ± 37.4 | 25 | 48.7 ± 38.5 | 18 | 56.5 ± 35.8 | 12 | 45.8 ± 32.7 | 9 | 55.6 ± 40.8 | 9 | 57.4 ± 42.6 | 16 | 38.5 ± 36.9 |
| Emotional function |  |  |  |  |  |  |  |  |  |  |  |  |  |  |
| Control group | 39 | 75.0 ± 24.9 | 28 | 79.2 ± 26.1 | 21 | 76.2 ± 23.9 | 15 | 88.9 ± 12.1 | 14 | 76.8 ± 18.3 | 11 | 83.3 ± 18.6 | 21 | 79.4 ± 19.1 |
| Experimental group | 37 | 73.6 ± 20.6 | 26 | 72.8 ± 25.1 | 18 | 71.3 ± 27.1 | 12 | 73.6 ± 31.3 | 9 | 75.9 ± 32.4 | 9 | 83.3 ± 33.3 | 16 | 75.5 ± 25.4 |
| Cognitive function |  |  |  |  |  |  |  |  |  |  |  |  |  |  |
| Control group | 39 | 68.4 ± 28.8 | 28 | 66.7 ± 38.2 | 21 | 73.8 ± 30.1 | 15 | 87.8 ± 17.2 | 14 | 83.3 ± 22.6 | 11 | 84.8 ± 24.1 | 21 | 76.2 ± 29.1 |
| Experimental group | 37 | 64.9 ± 24.5 | 26 | 60.9 ± 33.6 | 18 | 69.4 ± 33.9 | 12 | 79.2 ± 31.9 | 9 | 79.6 ± 33.1 | 9 | 74.1 ± 31.3 | 16 | 64.6 ± 30.4 |
| Social function |  |  |  |  |  |  |  |  |  |  |  |  |  |  |
| Control group | 39 | 64.1 ± 35.2 | 28 | 63.1 ± 35.8 | 21 | 67.5 ± 29.6 | 15 | 83.3 ± 15.4 | 14 | 65.5 ± 33.6 | 11 | 65.2 ± 38.3 | 21 | 61.9 ± 33.0 |
| Experimental group | 37 | 56.3 ± 30.5 | 26 | 55.8 ± 35.9 | 18 | 58.3 ± 34.9 | 12 | 61.1 ± 38.5 | 9 | 70.4 ± 43.1 | 9 | 72.2 ± 30.0 | 16 | 65.6 ± 27.5 |
| *C30 symptom scales* |  |  |  |  |  |  |  |  |  |  |  |  |  |  |
| Fatigue |  |  |  |  |  |  |  |  |  |  |  |  |  |  |
| Control group | 39 | 31.6 ± 24.3 | 28 | 34.9 ± 28.3 | 21 | 29.9 ± 28.4 | 15 | 23.7 ± 15.1 | 14 | 28.6 ± 27.8 | 11 | 26.3 ± 29.9 | 21 | 30.2 ± 27.5 |
| Experimental group | 37 | 35.7 ± 22.9 | 26 | 36.8 ± 26.6 | 18 | 31.5 ± 24.5 | 12 | 33.3 ± 30.3 | 9 | 29.6 ± 36.0 | 9 | 30.9 ± 38.0 | 16 | 43.1 ± 34.4 |
| Nausea/vomiting |  |  |  |  |  |  |  |  |  |  |  |  |  |  |
| Control group | 39 | 8.1 ± 12.6 | 28 | 12.5 ± 20.1 | 21 | 14.3 ± 17.7 | 15 | 1.1 ± 4.3 | 14 | 3.6 ± 9.6 | 11 | 4.5 ± 10.8 | 21 | 9.5 ± 22.7 |
| Experimental group | 37 | 12.2 ± 23.8 | 26 | 16.0 ± 24.3 | 18 | 13.9 ± 17.4 | 12 | 13.9 ± 25.5 | 9 | 5.6 ± 8.3 | 9 | 1.9 ± 5.6 | 16 | 8.3 ± 13.6 |
| Pain |  |  |  |  |  |  |  |  |  |  |  |  |  |  |
| Control group | 39 | 20.5 ± 29.2 | 28 | 16.1 ± 25.0 | 21 | 20.6 ± 21.7 | 15 | 16.7 ± 21.8 | 14 | 19.0 ± 23.4 | 11 | 16.7 ± 24.7 | 21 | 19.8 ± 30.6 |
| Experimental group | 37 | 23.9 ± 22.1 | 26 | 25.0 ± 25.1 | 18 | 19.4 ± 24.4 | 12 | 23.6 ± 34.4 | 9 | 27.8 ± 32.3 | 9 | 16.7 ± 33.3 | 16 | 32.3 ± 30.1 |
| Dyspnea |  |  |  |  |  |  |  |  |  |  |  |  |  |  |
| Control group | 39 | 12.8 ± 21.1 | 28 | 13.1 ± 21.0 | 21 | 11.1 ± 19.2 | 15 | 0 | 14 | 9.5 ± 15.6 | 11 | 3.0 ± 10.1 | 21 | 3.2 ± 14.5 |
| Experimental group | 37 | 11.7 ± 23.9 | 25 | 13.3 ± 27.2 | 18 | 14.8 ± 26.1 | 12 | 25.0 ± 37.9 | 9 | 11.1 ± 33.3 | 9 | 3.7 ± 11.1 | 16 | 10.4 ± 20.1 |
| Insomnia |  |  |  |  |  |  |  |  |  |  |  |  |  |  |
| Control group | 39 | 20.5 ± 32.1 | 28 | 15.5 ± 29.4 | 21 | 20.6 ± 30.7 | 15 | 13.3 ± 27.6 | 14 | 9.5 ± 20.4 | 11 | 18.2 ± 22.9 | 21 | 15.9 ± 22.7 |
| Experimental group | 37 | 20.7 ± 27.6 | 26 | 23.1 ± 29.5 | 18 | 20.4 ± 23.3 | 12 | 16.7 ± 26.6 | 9 | 11.1 ± 16.7 | 9 | 3.7 ± 11.1 | 16 | 18.8 ± 27.1 |
| Appetite loss |  |  |  |  |  |  |  |  |  |  |  |  |  |  |
| Control group | 39 | 20.5 ± 29.2 | 28 | 19.0 ± 29.3 | 21 | 20.6 ± 24.7 | 15 | 13.3 ± 16.9 | 14 | 23.8 ± 35.6 | 11 | 15.2 ± 31.1 | 21 | 12.7 ± 24.7 |
| Experimental group | 37 | 25.2 ± 31.8 | 26 | 26.9 ± 32.7 | 18 | 24.1 ± 22.3 | 12 | 19.4 ± 26.4 | 9 | 14.8 ± 24.2 | 9 | 14.8 ± 24.2 | 16 | 16.7 ± 29.8 |
| Constipation |  |  |  |  |  |  |  |  |  |  |  |  |  |  |
| Control group | 39 | 19.7 ± 29.3 | 28 | 23.8 ± 32.5 | 21 | 22.2 ± 33.9 | 15 | 13.3 ± 21.1 | 14 | 23.8 ± 20.4 | 11 | 24.2 ± 30.2 | 21 | 11.1 ± 19.2 |
| Experimental group | 36 | 21.3 ± 31.0 | 26 | 24.4 ± 27.6 | 18 | 16.7 ± 20.6 | 12 | 13.9 ± 26.4 | 9 | 7.4 ± 14.7 | 9 | 7.4 ± 14.7 | 16 | 10.4 ± 16.0 |
| Diarrhea |  |  |  |  |  |  |  |  |  |  |  |  |  |  |
| Control group | 39 | 6.8 ± 13.6 | 27 | 7.4 ± 23.3 | 21 | 12.7 ± 24.7 | 15 | 2.2 ± 8.6 | 14 | 11.9 ± 24.8 | 11 | 0 | 21 | 4.8 ± 12.0 |
| Experimental group | 36 | 14.8 ± 29.2 | 26 | 20.5 ± 21.2 | 18 | 22.2 ± 30.2 | 12 | 16.7 ± 26.6 | 9 | 11.1 ± 16.7 | 9 | 7.4 ± 14.7 | 16 | 18.8 ± 32.1 |
| Financial impact |  |  |  |  |  |  |  |  |  |  |  |  |  |  |
| Control group | 39 | 30.8 ± 34.5 | 28 | 26.2 ± 33.2 | 21 | 23.8 ± 28.2 | 15 | 11.1 ± 16.3 | 14 | 28.6 ± 28.8 | 11 | 27.3 ± 29.1 | 21 | 36.5 ± 34.8 |
| Experimental group | 37 | 35.1 ± 31.4 | 26 | 38.5 ± 33.6 | 18 | 35.2 ± 38.7 | 12 | 30.6 ± 41.3 | 9 | 18.5 ± 37.7 | 9 | 22.2 ± 37.3 | 16 | 29.2 ± 34.2 |
| *BN20 symptom scales* |  |  |  |  |  |  |  |  |  |  |  |  |  |  |
| Future uncertainty |  |  |  |  |  |  |  |  |  |  |  |  |  |  |
| Control group | 39 | 32.9 ± 26.4 | 28 | 27.1 ± 22.4 | 21 | 25.8 ± 19.9 | 15 | 12.8 ± 14.0 | 14 | 22.6 ± 18.9 | 11 | 15.2 ± 15.3 | 21 | 22.2 ± 23.3 |
| Experimental group | 37 | 36.7 ± 24.5 | 26 | 32.7 ± 24.4 | 18 | 33.8 ± 25.2 | 12 | 28.5 ±27.2 | 9 | 25.0 ± 30.3 | 9 | 16.7 ± 26.0 | 16 | 28.1 ± 25.8 |
| Visual disorder |  |  |  |  |  |  |  |  |  |  |  |  |  |  |
| Control group | 39 | 18.2 ± 24.9 | 28 | 16.7 ± 23.1 | 21 | 9.0 ± 12.0 | 15 | 6.7 ± 12.5 | 14 | 12.7 ± 13.0 | 11 | 11.1 ± 14.1 | 21 | 23.3 ± 30.2 |
| Experimental group | 37 | 24.3 ± 27.9 | 26 | 28.6 ± 33.9 | 18 | 27.8 ± 31.5 | 12 | 25.0 ± 34.2 | 9 | 17.3 ± 33.4 | 9 | 17.3 ± 33.4 | 16 | 16.0 ± 28.1 |
| Motor dysfunction |  |  |  |  |  |  |  |  |  |  |  |  |  |  |
| Control group | 39 | 32.2 ± 36.8 | 28 | 31.0 ± 36.0 | 21 | 23.8 ± 31.3 | 15 | 9.6 ± 17.8 | 14 | 20.6 ± 26.8 | 11 | 11.1 ± 12.2 | 21 | 29.6 ± 34.8 |
| Experimental group | 37 | 36.0 ± 33.4 | 26 | 46.2 ± 37.8 | 18 | 38.3 ± 29.1 | 12 | 47.2 ± 32.2 | 9 | 32.1 ± 34.9 | 9 | 33.3 ± 33.8 | 16 | 53.5 ± 32.3 |
| Communication deficit |  |  |  |  |  |  |  |  |  |  |  |  |  |  |
| Control group | 39 | 24.5 ± 31.3 | 28 | 27.8 ± 34.8 | 21 | 26.5 ± 37.4 | 15 | 21.5 ± 37.0 | 14 | 21.4 ± 31.6 | 11 | 21.2 ± 33.5 | 21 | 21.2 33.1 |
| Experimental group | 37 | 33.2 ± 31.4 | 26 | 39.3 ± 37.6 | 18 | 29.6 ± 27.5 | 12 | 26.9 ± 25.7 | 9 | 17.3 ± 22.3 | 9 | 13.6 ± 15.5 | 16 | 27.1 ± 24.0 |
| Headache |  |  |  |  |  |  |  |  |  |  |  |  |  |  |
| Control group | 39 | 22.2 ± 29.0 | 28 | 17.9 ± 26.4 | 21 | 17.5 ± 22.7 | 15 | 17.8 ± 27.8 | 14 | 21.4 ± 28.1 | 11 | 21.2 ± 37.3 | 21 | 25.4 ± 37.9 |
| Experimental group | 37 | 27.0 ± 27.0 | 26 | 25.6 ± 28.8 | 18 | 18.5 ± 20.5 | 12 | 19.4 ± 22.3 | 9 | 11.1 ± 23.6 | 9 | 14.8 ± 33.8 | 16 | 16.7 ± 27.2 |
| Seizures |  |  |  |  |  |  |  |  |  |  |  |  |  |  |
| Control group | 39 | 4.3 ± 11.3 | 28 | 8.3 ± 19.5 | 21 | 3.2 ± 10.0 | 15 | 2.2 ± 8.6 | 14 | 0 | 11 | 0 | 21 | 1.6 ± 7.3 |
| Experimental group | 36 | 7.4 ± 16.2 | 26 | 12.8 ± 28.4 | 18 | 14.8 ± 28.5 | 12 | 16.7 ± 33.3 | 9 | 11.1 ± 33.3 | 9 | 3.7 ± 11.1 | 16 | 0 |
| Drowsiness |  |  |  |  |  |  |  |  |  |  |  |  |  |  |
| Control group | 39 | 30.8 ± 28.0 | 28 | 34.5 ± 32.1 | 21 | 30.2 ± 23.3 | 15 | 24.4 ± 19.8 | 14 | 33.3 ± 18.5 | 11 | 30.3 ± 23.4 | 21 | 36.5 ± 27.7 |
| Experimental group | 37 | 36.9 ± 27.0 | 26 | 46.2 ± 32.8 | 18 | 37.0 ± 27.7 | 12 | 36.1 ± 30.0 | 9 | 37.0 ± 35.1 | 9 | 37.0 ± 35.1 | 16 | 45.8 ± 29.5 |
| Hair loss |  |  |  |  |  |  |  |  |  |  |  |  |  |  |
| Control group | 39 | 13.7 ± 26.2 | 28 | 11.9 ± 26.0 | 21 | 4.8 ± 12.0 | 15 | 2.2 ± 8.6 | 14 | 2.4 ± 8.9 | 11 | 3.0 ± 10.1 | 21 | 4.8 ± 12.0 |
| Experimental group | 36 | 14.8 ± 21.7 | 26 | 10.3 ± 22.6 | 18 | 13.0 ± 20.3 | 12 | 19.4 ± 33.2 | 9 | 7.4 ± 14.7 | 9 | 0 | 16 | 10.4 ± 23.5 |
| Itchy skin |  |  |  |  |  |  |  |  |  |  |  |  |  |  |
| Control group | 39 | 13.7 ± 21.2 | 28 | 7.1 ± 13.9 | 21 | 9.5 ± 15.4 | 15 | 4.4 ± 11.7 | 14 | 7.1 ± 19.3 | 11 | 6.1 ± 20.1 | 21 | 3.2 ± 10.0 |
| Experimental group | 36 | 15.7 ± 20.3 | 26 | 15.4 ± 28.6 | 18 | 18.5 ± 30.7 | 12 | 13.9 ± 26.4 | 9 | 7.4 ± 14.7 | 9 | 0 | 16 | 10.4 ± 16.0 |
| Weakness of legs |  |  |  |  |  |  |  |  |  |  |  |  |  |  |
| Control group | 39 | 38.5 ± 37.1 | 28 | 35.7 ± 38.4 | 21 | 22.2 ± 35.5 | 15 | 17.8 ± 30.5 | 14 | 26.2 ± 26.7 | 11 | 15.2 ± 17.4 | 21 | 38.1 ± 32.1 |
| Experimental group | 36 | 40.7 ± 33.9 | 26 | 56.4 ± 39.7 | 18 | 40.7 ± 31.4 | 12 | 50.0 ± 33.3 | 9 | 40.7 ± 40.1 | 9 | 51.9 ± 37.7 | 16 | 58.3 ± 33.3 |
| Bladder control |  |  |  |  |  |  |  |  |  |  |  |  |  |  |
| Control group | 39 | 14.5 ± 30.4 | 28 | 25.0 ± 35.9 | 21 | 15.9 ± 30.9 | 15 | 17.8 ± 30.5 | 14 | 21.4 ± 36.1 | 11 | 18.2 ± 31.1 | 21 | 11.1 ± 24.3 |
| Experimental group | 36 | 18.5 ± 24.5 | 26 | 25.6 ± 35.7 | 18 | 20.4 ± 30.5 | 12 | 19.4 ± 36.1 | 9 | 14.8 ± 33.8 | 9 | 14.8 ± 33.8 | 16 | 20.8 ± 29.5 |

Abbreviations: n, number; sd, standard deviation
